# Epidemiology of birth defects based on a birth defects surveillance system in southwestern China and the associated risk factors

**DOI:** 10.3389/fped.2023.1165477

**Published:** 2023-07-21

**Authors:** Zhenren Peng, Jie Wei, Biyan Chen, Xiuning Huang, Pengshu Song, Lifang Liang, Jiajia He, Baoying Feng, Ting Que, Jie Qin, Yu'an Xie, Xiaoxia Qiu, Hongwei Wei, Sheng He

**Affiliations:** ^1^ Birth Defects Research Laboratory, Maternal and Child Health Hospital of Guangxi Zhuang Autonomous Region, Nanning, China; ^2^ Birth Defects Research Laboratory, Birth Defects Prevention and Control Institute of Guangxi Zhuang Autonomous Region, Nanning, China; ^3^ Birth Defects Research Laboratory, Guangxi Key Laboratory of Reproductive Health and Birth Defect Prevention, Nanning, China; ^4^ Birth Defects Research Laboratory, Guangxi Key Laboratory of Birth Defects Research and Prevention, Nanning, China

**Keywords:** birth defects, perinatal infants, pregnant women, epidemiology, risk factors

## Abstract

**Background:**

Birth defects (BDs) are associated with many potential risk factors, and its causes are complex.

**Objectives:**

This study aimed to explore the epidemiological characteristics of BDs in Guangxi of China and the associated risk factors of BDs.

**Methods:**

BDs data of perinatal infants (PIs) were obtained from the Guangxi birth defects monitoring network between 2016 and 2020. Univariate Poisson regression was used to calculate the prevalence-rate ratios (PRR) to explore the changing trends of BDs prevalence by year and the correlation between the regarding of characteristics of BDs (including infant gender, maternal age, and quarter) and BDs. Clinical characteristics of PIs with BDs and general characteristics of their mothers were documented, and Spearman correlation analysis was used to explore the potential associated risk factors of BDs.

**Results:**

Between 2016 and 2020, 44,146 PIs with BDs were monitored, with an overall BDs prevalence of 121.71 (95% CI: 120.58–122.84) per 10,000 PIs, showing a significant increase trend (PRR = 1.116, 95% CI: 1.108–1.123), especially the prevalence of congenital heart defects (CHDs) that most significantly increased (PRR = 1.300, 95% CI: 1.283–1.318). The 10 most common BDs were CHDs, polydactyly, congenital talipes equinovarus, other malformation of external ear, syndactyly, hypospadias, cleft lip with cleft palate, cleft lip, hemoglobin Bart's hydrops fetalis syndrome (BHFS), and congenital atresia of the rectum and anus. BDs were positively correlated with pregnant women's age (*R* = 0.732, *P* < 0.01) and education level (*R* = 0.586, *P* < 0.05) and having pre-gestational diabetes mellitus (PGDM)/gestational diabetes mellitus (GDM) (*R* = 0.711, *P* < 0.01), while when the pregnant women had a family history of a dead fetus (*R* = −0.536, *P* < 0.05) and a birth of a fetus with BDs (*R* = −0.528, *P* < 0.05) were negatively correlated with BDs.

**Conclusion:**

A significant increase in the prevalence of BDs was detected between 2016 and 2020 in Guangxi, especially the prevalence of CHDs that most significantly increased. Older maternal age, higher maternal education level, and having PGDM before pregnancy or GDM in early pregnancy were the risk factors for BDs.

## Introduction

Birth defects (BDs) or congenital anomalies are defined as any structural or functional abnormalities, including metabolic disorders, which occur during intrauterine pregnancy mostly occurring in the first 3 months of pregnancy (1–3). The World Health Organization (WHO) reports that BDs are the main causes of spontaneous abortions for pregnant women, stillbirths for perinatal infants (PIs), and death and disability for children under 5 years old (3). Approximately 3.00%–6.00% of infants are born with serious BDs, and more than 3.3 million children die of BDs in the world every year (4, 5), especially that BDs are the main causes of the loss of disability-adjusted life years (DALYs) for children under 5 years old (6–9). In China, the prevalence of BDs is about 5.60%, children's life and quality of life can be seriously affected by BDs, and BDs can bring great pain and financial burden to children and their families (10).

The causes of BDs are complex. It is well known that approximately 65.00%–70.00% of BDs are ascribed to complex genetics with unknown factors (2). Several researchers have also shown that the potential associated risk factors of BDs included maternal age, gravidity, parity, monthly income of the family, education level of pregnant women (11), household registration, history of miscarriages, family history of BDs, infection, taking medicine, pesticide exposure, and single/twin pregnancy (12). Therefore, how to effectively prevent BDs has become very urgent and important. Some scholars believed that the key to the prevention of BDs lies in the effective detection of BDs and the full understanding of BDs (13). In another study, some other scholars believed that pediatricians play a key role in the prevention of BDs and then propose many effective measures for the prevention of BDs, for example, pregnancy planning, intake of folic acid supplements before and during pregnancy, and no prenatal exposure to teratogenic agents (14). It is well known that it is widely different in time and associated risk factors for the prevalence of BDs vary; to describe the epidemiology of BDs and to understand the associated risk factors of BDs are important to provide epidemiological evidence for putting forward effective measures to prevent BDs (15, 16).

Guangxi is a western economic province located in southwestern China. In 2021, the permanent resident population of Guangxi is 50.37 million, of which the population of Han nationality is 31.46 million, accounting for 62.45%; the population of ethnic groups is 18.91 million, accounting for 37.55%, of which the population of Zhuang nationality is 15.79 million, accounting for 31.35%; the birth population is 0.49 million; and the birth rate is 9.68% (17). In Guangxi, BDs are still a big public health problem. Previous studies and other studies have shown that the prevalence of BDs in Guangxi is about 1% (18–21); however, these studies only briefly described the epidemiology of BDs, and the study of associated risk factors of BDs in Guangxi is relatively reported. Therefore, describing the epidemiology of BDs and understanding the associated risk factors of BDs in Guangxi are also necessary. The aims of this study were to explore the epidemiological characteristics of BDs in Guangxi between 2016 and 2020 and then to explore the associated risk factors of BDs. It is believed that this study will provide a reference for revealing the potential associated risk factors of BDs based on the BDs surveillance system.

## Methods

### Data source and data collection

Guangxi birth defects monitoring network (GXBDMN) is a hospital-based BDs surveillance system. Early fetuses of <28 weeks of gestation and PIs between 28 weeks of gestation and 7 days after delivery born in hospitals were all included in this surveillance system, including live birth, stillbirth, dead fetuses, and fetuses with BDs. According to “Maternal and Child Health Surveillance of China Work Manual (2021 Edition)” and “Guangxi Birth Defects Hospital Monitoring Scheme,” when a fetus with BDs was born from induced labor in a monitoring hospital, regardless of gestational age, the “Medical Institution Birth Defects Fetus Registration Card” was used to record BDs case data. However, when calculating the prevalence of BDs for perinatal infants, only fetuses of ≥28 weeks of gestation with BDs are included. The monitoring content of BDs includes the time, region, and population distribution of major BDs and clinical data as well as the potential associated risk factors for BDs.

The International Statistical Classification of Diseases and Related Health Problems, Tenth Revision (ICD-10) is the diagnostic criteria for BDs. The screening and diagnostic methods for BDs mainly included high-risk factor screening, clinical observation and physical examination, and auxiliary examinations. The high-risk factor screening included family history, environmental teratogenic factors, and pregnancy history (early pregnancy, high-risk pregnancy, and high-risk neonatal history). Furthermore, the auxiliary examinations included ultrasound diagnosis, x-ray examination, autopsy and pathological section observation, chromosome abnormality detection, and blood biochemical and immunological examinations. The GXBDMN was mainly focusing on monitoring 25 types of common structural abnormalities, chromosomal abnormalities, and a small number of genetic and metabolic diseases in PIs. The 25 types of BDs were anencephaly, spina bifida, encephalocele, congenital hydrocephalus, cleft palate, cleft lip, cleft lip with cleft palate, microtia, other malformation of external ear, congenital esophageal atresia, congenital atresia of the rectum and anus, hypospadias, bladder exstrophy, congenital talipes equinovarus, polydactyly, syndactyly, limp reduction defects, congenital diaphragmatic hernia, omphalocele, gastroschisis, conjoined twins, Down syndrome (DS), congenital heart defects (CHDs), hemoglobin Bart's hydrops fetalis syndrome (BHFS), and other types of BDs (such as polycystic kidney, lymphangioma, hemangioma, and teratoma).

In this study, the study population was those PIs who were diagnosed with BDs and their mothers, who were all enrolled in GXBDMN between 2016 and 2020. The basic socio-demographic data and clinical information of this study population were all obtained from GXBDMN. In this study, the prevalence of BDs was defined as the number of PIs that were diagnosed with BDs per 10,000 PIs.

In addition, data cleaning was conducted before starting the statistics analysis. The prevalence of other types of BDs (such as polycystic kidney, lymphangioma, hemangioma, and teratoma) was not calculated and separately displayed in this study, but it was taken into account when calculating the total prevalence of BDs. Moreover, when calculating the BDs prevalence of PIs in different genders, PIs of unknown gender were not separately listed. The selection of potential covariates, such as clinical characteristics of PIs with BDs and general characteristics of their mothers, and potential associated risk factors of BDs were mainly based on the “Medical Institution Birth Defects Fetus Registration Card,” which was used to record data on BDs cases.

### Statistics analysis

The total prevalence of BDs was calculated by the year, infant gender, maternal age, and quarter. The prevalence of each type of BDs was also separately calculated by year and ranked by total prevalence in descending order. The 95% confidence intervals (CI) of the total prevalence of BDs and the prevalence of each type of BDs were all separately calculated by each year and across all years.

Univariate and multivariate Poisson regressions are often applied to evaluate the association between the outcome variable and covariates, especially used to evaluate the temporal trends of BDs prevalence (22–24). In this study, the univariate Poisson regression was used to calculate the prevalence-rate ratios (PRR) and 95% CI of PRR to explore the changing trends of BDs prevalence by year and the regarding of characteristics of BDs, including infant gender (female as a reference), maternal age (20–24 years as a reference), and quarters (the fourth quarter as a reference). In this study, the PRR was a crude value without any adjustment of testing. The univariate Poisson regression was performed within the Stata MP 16.0 software (StataCorp LLC, Texas, USA). The analysis path order was statistics, count outcomes, and Poisson regression. In the “model” analysis module, the dependent valuable was set to the number of BDs, the independent valuable was set to years (infant gender, maternal age, or quarters), and the explore valuable was set to the number of PIs. In the “reporting” analysis module, the report of incidence-rate ratios was set to 95% CI. In this study, the PRR was equivalent to the report of incidence-rate ratios. The PRR could indicate the magnitude of change in BDs prevalence, which would not be a negative value. If the PRR was <1 and had statistical significance, it indicated a decrease in BDs prevalence, while if the PRR was >1 and had statistical significance, it indicated an increase in BDs prevalence.

The clinical characteristics of PIs with BDs and their mothers were described as follows: PIs’ gestational age, number of fetuses, clinical outcomes, induction of labor after diagnosis of defects, diagnostic methods, and diagnosis time of malformation and the mother's age, ethnic group, education level, monthly income per capita (RMB) of the family, gravidity, and parity.

Pearson correlation analysis and Spearman correlation analysis are usually used to explore potential risk factors of BDs (25, 26). Considering the non-normal distribution model of BDs data in this study, Spearman correlation analysis was used to explore the potential associated risk factors of BDs. In this study, the potential associated risk factors of BDs were documented, including pregnant women's basic socio-demographic (age, education level, and monthly income per capita of the family), medications in early pregnancy (antibiotic use, contraceptive drug use, and sedative use), illness in early pregnancy [fever, viral infection, and pre-gestational diabetes mellitus (PGDM)/gestational diabetes mellitus (GDM)], exposure to harmful substances in early pregnancy (alcohol use and pesticide/radiation/chemical exposure), and family history (gravidity, parity, dead fetus, spontaneous abortions, birth of a fetus with BDs, and consanguineous marriage). The Spearman correlation analysis was conducted within the Stata MP 16.0 software. The dependent variable and independent variable were BDs prevalence and the potential associated risk factors of BDs, respectively. The running command of Spearman correlation analysis was “Spearman, stats(rho p).” The *R*-value could indicate the strength of the correlation between BDs prevalence and the potential risk factors of BDs. If the *R*-value was <0 and had statistical significance, it indicated a negative correlation between BDs prevalence and the potential risk factors of BDs, while if the *R*-value was >0 and had statistical significance, it indicated a positive correlation between BDs prevalence and potential risk factors of BDs.

In this study, all statistical analyses were conducted within the Stata MP 16.0 software, with a significance level of *P* < 0.05.

## Results

### Trends of total BDs prevalence and the perspective of maternal characteristics

Between 2016 and 2020, 3,627,108 PIs were monitored by GXBDMN, of which 44,146 PIs were diagnosed with BDs, resulting in an overall BDs prevalence of 121.71 (95% CI: 120.58–122.84) per 10,000 PIs; 3,607,925 live birth PIs were monitored by GXBDMN, of which 39,259 live birth PIs were diagnosed with BDs, resulting in an overall live birth BDs prevalence of 108.81 (95% CI: 107.74–109.88) per 10,000 live birth PIs. The univariate Poisson regression results showed that the prevalence of BDs significantly increased during the study period (PRR = 1.116, 95% CI: 1.108–1.123). The prevalence of BDs in male PIs was significantly higher than that in female PIs (PRR = 1.289, 95% CI: 1.265–1.314), and the prevalence of BDs in male and female PIs were 135.76 (95% CI: 134.13–137.40) and 105.29 (95% CI: 103.76–106.83) per 10,000 PIs, respectively. The prevalence of live birth BDs in male live birth PIs was also significantly higher than that in female live birth PIs (PRR = 1.320, 95% CI: 1.294–1.347), and the prevalence of live birth BDs in male and female live birth PIs were 122.64 (95% CI: 121.08–124.20) and 92.91 (95% CI: 91.47–94.36) per 10,000 live birth PIs, respectively. The prevalence of BDs in mothers aged 20–24 years was lower than that in other age groups of mothers, and the prevalence of BDs in mothers aged 35 years or above was the highest (PRR = 1.389, 95% CI: 1.346–1.433). The prevalence of BDs in mothers aged 35 years or above and aged 20–24 years were 149.74 (95% CI: 146.51–152.96) and 107.84 (95% CI: 105.39–110.28) per 10,000 PIs, respectively. The prevalence of BDs in the fourth quarter (October, November, December) was lower than that in other quarters, and the prevalence of BDs in the second quarter (April, May, June) was the highest (PRR = 1.082, 95% CI: 1.054–1.111). The prevalence of total BDs analyzed over the years and the perspective of maternal characteristics are shown in Table 1. And the prevalence of total live birth BDs analyzed over the years and the perspective of infant gender are shown in Table 2.

**Table 1:** Prevalence of total birth defects analyzed over the years and the perspective of maternal characteristics.

Variables	Number of PIs	Number of BDs	BDs prevalence (95% CI)	PRR (95% CI)
Years				
2016	855,720	8,819	103.06 (100.92–105.20)	1.116 (1.108–1.123)
2017	826,295	8,746	105.85 (103.64–108.05)	
2018	713,609	8,530	119.53 (117.01–122.05)	
2019	653,593	9,073	138.82 (135.98–141.65)	
2020	577,891	8,978	155.36 (152.17–158.55)	
Total	3,627,108	44,146	121.71 (120.58–122.84)	
Infant gender				
Male	1,924,367	26,126	135.76 (134.13–137.40)	1.289 (1.265–1.314)
Female	1,701,658	17,917	105.29 (103.76–106.83)	Reference
Maternal age, years				
<20	192,861	2,280	118.22 (113.40–123.04)	1.096 (1.046–1.149)
20–24	685,743	7,395	107.84 (105.39–110.28)	Reference
25–29	1,200,737	13,674	113.88 (111.98–115.78)	1.056 (1.027–1.086)
30–34	1,001,806	12,622	125.99 (123.81–128.18)	1.168 (1.135–1.202)
≥35	545,961	8,175	149.74 (146.51–152.96)	1.389 (1.346–1.433)
Quarter				
First quarter	880,818	10,637	120.76 (118.48–123.04)	1.028 (1.001–1.055)
Second quarter	820,652	10,438	127.19 (124.77–129.62)	1.082 (1.054–1.111)
Third quarter	905,308	11,082	122.41 (120.15–124.68)	1.042 (1.015–1.069)
Fourth quarter	1,020,330	11,989	117.50 (115.41–119.59)	Reference

BDs, firth defects; PIs, perinatal infants; CI, confidence intervals; PRR, prevalence-rate ratios; the first quarter includes January, February, and March; the second quarter includes April, May, and June; the third quarter includes July, August, and September; the fourth quarter includes October, November, and December.

**Table 2:** Prevalence of total live birth defects analyzed over the years and the perspective of infant gender.

Variables	Number of live birth PIs	Number of live birth BDs	Live birth BDs prevalence (95% CI)	PRR (95% CI)
Years				
2016	850,239	7,492	88.12 (86.13–90.10)	1.143 (1.135–1.151)
2017	821,933	7,547	91.82 (89.76–93.88)	
2018	710,080	7,593	106.93 (104.54–109.32)	
2019	650,456	8,253	126.88 (124.16–129.60)	
2020	575,217	8,374	145.58 (142.48–148.68)	
Total	3,607,925	39,259	108.81 (107.74–109.88)	
Infant gender				
Male	1,914,756	23,483	122.64 (121.08–124.20)	1.320 (1.294–1.347)
Female	1,693,095	15,731	92.91 (91.47–94.36)	Reference

BDs, birth defects; PIs, perinatal infants; CI, confidence intervals; PRR, prevalence-rate ratios; the first quarter includes January, February, and March; the second quarter includes April, May, and June; the third quarter includes July, August, and September; the fourth quarter includes October, November, and December.

### Clinical characteristics of PIs with BDs and general characteristics of their mothers

Table 3 shows the clinical characteristics of PIs with BDs. As shown in Table 3, most PIs with BDs were full-term infants (37–42 weeks), accounting for 77.94%; followed by premature infants (32–37 weeks) and extremely premature infants (28–32 weeks), accounting for 14.75% and 6.81%, respectively; post-term infants (≥42 weeks) were the least, only accounting for 0.49%. Among these PIs, most of them were single births, accounting for 96.66%, and twins and multiple births were only accounting for 3.32% and 0.02%, respectively. In clinical outcomes, most PIs with BDs were live births, accounting for 88.93%; followed by dead fetuses, accounting for 10.17%; 0.63% of PIs with BDs died within 7 days after delivery; and the stillbirths accounted for 0.27%. Moreover, 10.17% of PIs involved induction of labor after diagnosis of BDs. Approximately 57.86% and 32.78% of PIs were, respectively, diagnosed with BDs by the methods of clinical diagnosis after birth and prenatal ultrasound diagnosis. In diagnosis time of malformation, 80.16% and 19.84% PIs were, respectively, diagnosed with BDs within 7 days after delivery and in antenatal care.

**Table 3:** Clinical characteristics of perinatal infants with birth defects.

Variables	Number of BDs	Percentage
Total number of BDs	44,146	100.00
Gestational age, weeks		
28–32 (extremely premature infant)	3,008	6.81
32–37 (premature infant)	6,513	14.75
37–42 (full-term infant)	34,408	77.94
≥42 (post-term infant)	217	0.49
Number of fetuses		
Single	42,670	96.66
Twins	1,466	3.32
Multifetal	10	0.02
Clinical outcomes		
Live birth	39,259	88.93
Dead fetus	4,488	10.17
Stillbirth	121	0.27
Death within 7 days	278	0.63
Induction of labor after diagnosis of defects		
Yes	4,488	10.17
No	39,658	89.83
Diagnostic method		
Clinical diagnosis after birth	25,544	57.86
Prenatal ultrasound diagnosis	14,472	32.78
Clinical diagnosis after birth and prenatal ultrasound diagnosis	1,777	4.03
Autopsy	11	0.02
Unknown	2,342	5.31
Diagnosis time of malformation		
Antenatal	8,760	19.84
Within 7 days after delivery	35,386	80.16

BDs, birth defects.

Table 4 shows the general characteristics of pregnant women whose PIs were diagnosed with BDs. The average maternal age was 29.17 ± 5.90 years. Approximately 68.39% and 26.05% of pregnant women were Han nationality and Zhuang nationality, respectively, totaling 94.44%, while only 5.56% of pregnant women were Yao nationality, Miao nationality, and other nationalities. Approximately 40.87% and 37.57% of pregnant women had a junior middle school degree and a college degree or above, respectively, totaling 78.44% and 15.09% of pregnant women had a senior middle school degree, while only 6.46% of pregnant women were illiterate and had an elementary school degree. Approximately 30.48%, 28.46%, and 20.27% of pregnant women's family monthly income per capita were ≥8,000 RMB; 4,000–8,000 RMB; and 2,000–4,000 RMB, respectively, totaling 79.21%, while only 16.15% and 4.64% of pregnant women's family monthly income per capita were <1,000 RMB and 1,000–2,000 RMB, respectively. Only 24.96% of pregnant women were first-time gravidity, and 28.71% and 22.72% of pregnant women had two and three gravidities, respectively, while 13.22% and 10.39% of pregnant women's gravidity were 4 and ≥5, respectively. Approximately 41.83% and 40.90% of pregnant women's parity were 2 and 1, respectively, totaling 82.72%, and 12.41% and 4.15% of pregnant women's parity were 3 and ≥4, respectively, while only 0.72% of pregnant women had no history of parity.

**Table 4:** General characteristics of pregnant women whose perinatal infants were diagnosed with birth defects.

Variables	Number of BDs	Percentage
Total number of BDs	44,146	100.00
Ethnic group		
Han	30,191	68.39
Zhuang	11,501	26.05
Yao	1,252	2.84
Miao	447	1.01
Others	755	1.71
Education level		
Illiteracy	465	1.05
Elementary school	2,389	5.41
Junior middle school	18,043	40.87
Senior middle school	6,662	15.09
College degree or above	16,587	37.57
Family monthly income per capita, RMB		
<1,000	7,129	16.15
1,000∼2,000	2,049	4.64
2,000∼4,000	8,948	20.27
4,000∼8,000	12,563	28.46
≥8,000	13,457	30.48
Gravidity		
1	11,018	24.96
2	12,675	28.71
3	10,031	22.72
4	5,836	13.22
≥5	4,586	10.39
Parity		
0	316	0.72
1	18,054	40.90
2	18,465	41.83
3	5,477	12.41
≥4	1,834	4.15

BDs, birth defects.

### Prevalence and trends of each type of BDs

Table 5 shows the prevalence and trends of each type of BDs in Guangxi between 2016 and 2020. As shown in Table 5, during the 5-year study period, the 10 most common BDs were CHDs, polydactyly, congenital talipes equinovarus, other malformation of external ear, syndactyly, hypospadias, cleft lip with cleft palate, cleft lip, BHFS, and congenital atresia of the rectum and anus, with a total prevalence of 29.82 (95% CI: 29.26–30.38), 23.25 (95% CI: 22.76–23.75), 6.82 (95% CI: 6.55–7.09), 6.17 (95% CI: 5.91–6.42), 5.85 (95% CI: 5.60–6.10), 5.34 (95% CI: 5.11–5.58), 3.92 (95% CI: 3.71–4.12), 3.10 (95% CI: 2.92–3.28), 2.42 (95% CI: 2.26–2.58), and 1.98 (95% CI: 1.84–2.13) per 10,000 PIs, respectively. Between 2016 and 2020, among the 10 most common BDs, the prevalence of BHFS (PRR = 0.764, 95% CI: 0.726–0.804) and cleft lip with cleft palate (PRR = 0.915, 95% CI: 0.881–0.951) significantly decreased, while cleft lip (PRR = 0.980, 95% CI: 0.940–1.022) and congenital atresia of the rectum and anus (PRR = 1.004, 95% CI: 0.991–1.100) remained stable, and the others significantly increased through the study period, especially that the prevalence of CHDs (PRR = 1.300, 95% CI: 1.283–1.318) most significantly increased. Furthermore, the prevalence of CHDs increased from 20.67 per 10,000 PIs in 2016 to 54.01 per 10,000 PIs in 2020, while the prevalence of polydactyly (PRR = 1.066, 95% CI: 1.050–1.082), congenital talipes equinovarus (PRR = 1.069, 95% CI: 1.039–1.099), other malformation of external ear (PRR = 1.252, 95% CI: 1.215–1.289), syndactyly (PRR = 1.154, 95% CI: 1.119–1.189), and hypospadias (PRR = 1.086, 95% CI: 1.052–1.121) also significantly increased.

**Table 5:** Prevalence and trends of each type of birth defects in Guangxi, 2016–2020 (per 10,000 perinatal infants).

Types of BDs	Year					Total (95% CI)	Rank	PRR (95% CI)
	2016	2017	2018	2019	2020			
CHDs	20.67	19.97	27.13	35.82	54.01	29.82 (29.26–30.38)	1	1.300 (1.283–1.318)
Polydactyly	20.72	21.51	23.93	25.47	26.15	23.25 (22.76–23.75)	2	1.066 (1.050–1.082)
Congenital talipes equinovarus	6.12	5.69	7.67	8.00	7.09	6.82 (6.55–7.09)	3	1.069 (1.039–1.099)
Other malformation of external ear	1.51	6.80	8.41	8.17	7.13	6.17 (5.91–6.42)	4	1.252 (1.215–1.289)
Syndactyly	3.91	5.49	6.33	7.11	7.18	5.85 (5.60–6.10)	5	1.154 (1.119–1.189)
Hypospadias	4.41	5.36	5.34	5.37	6.68	5.34 (5.11–5.58)	6	1.086 (1.052–1.121)
Cleft lip with cleft palate	4.63	4.25	3.55	3.46	3.37	3.92 (3.71–4.12)	7	0.915 (0.881–0.951)
Cleft lip	3.10	3.28	3.08	3.14	2.82	3.10 (2.92–3.28)	8	0.980 (0.940–1.022)
BHFS	3.72	2.78	2.00	1.74	1.25	2.42 (2.26–2.58)	9	0.764 (0.726–0.804)
Congenital atresia of the rectum and anus	1.57	2.20	2.03	2.30	1.87	1.98 (1.84–2.13)	10	1.044 (0.991–1.100)
Cleft palate	1.72	1.68	1.61	2.02	2.92	1.94 (1.79–2.08)	11	1.137 (1.079–1.199)
Microtia	1.74	1.84	1.91	2.00	1.51	1.81 (1.67–1.94)	12	0.989 (0.937–1.045)
Limp reduction defects	1.23	1.39	1.33	1.21	1.11	1.26 (1.15–1.38)	13	0.971 (0.909–1.037)
DS	1.19	1.13	1.28	1.27	0.97	1.17 (1.06–1.28)	14	0.980 (0.915–1.049)
Congenital hydrocephalus	1.30	1.13	1.15	1.09	1.07	1.16 (1.04–1.27)	15	0.957 (0.893–1.025)
Omphalocele	0.49	0.45	0.60	0.55	0.57	0.53 (0.45–0.60)	16	1.053 (0.952–1.165)
Congenital diaphragmatic hernia	0.30	0.42	0.35	0.37	0.22	0.34 (0.28–0.40)	17	0.950 (0.836–1.079)
Congenital esophageal atresia	0.50	0.18	0.28	0.40	0.29	0.33 (0.27–0.39)	18	0.931 (0.818–1.059)
Spina bifida	0.39	0.29	0.34	0.38	0.22	0.33 (0.27–0.39)	19	0.936 (0.822–1.066)
Gastroschisis	0.18	0.35	0.14	0.21	0.28	0.23 (0.18–0.28)	20	1.024 (0.879–1.193)
Anencephaly	0.21	0.24	0.29	0.17	0.12	0.21 (0.16–0.26)	21	0.900 (0.764–1.060)
Encephalocele	0.18	0.08	0.11	0.05	0.03	0.10 (0.06–0.13)	22	0.689 (0.526–0.902)
Bladder exstrophy	0.04	0.01	0.00	0.03	0.03	0.02 (0.01–0.04)	23	1.039 (0.634–1.704)
Conjoined twins	0.02	0.02	0.00	0.02	0.00	0.01 (0.00–0.03)	24	0.623 (0.293–1.326)

BDs, birth defects; CI, confidence intervals; PRR, prevalence-rate ratios; CHDs, congenital heart defects; BHFS, hemoglobin Bart’s hydrops fetalis syndrome; DS, Down syndrome.

In addition, among all types of BDs, the prevalence of encephalocele (PRR = 0.689, 95% CI: 0.526–0.902) most significantly decreased through the study period. The prevalence of encephalocele decreased from 0.18 per 10,000 PIs in 2016 to 0.03 per 10,000 PIs in 2020, with a total prevalence of 0.10 (95% CI: 0.06–0.13) per 10,000 PIs, while the prevalence of cleft palate (PRR = 1.137, 95% CI: 1.079–1.199), microtia (PRR = 0.989, 95% CI: 0.937–1.045), limp reduction defects (PRR = 0.971, 95% CI: 0.909–1.037), DS (PRR = 0.980, 95% CI: 0.915–1.049), congenital hydrocephalus (PRR = 0.957, 95% CI: 0.893–1.025), omphalocele (PRR = 1.053, 95% CI: 0.952–1.165), congenital diaphragmatic hernia (PRR = 0.950, 95% CI: 0.836–1.079), congenital esophageal atresia (PRR = 0.931, 95% CI: 0.818–1.059), spina bifida (PRR = 0.936, 95% CI: 0.822–1.066), gastroschisis (PRR = 1.024, 95% CI: 0.879–1.193), anencephaly (PRR = 0.900, 95% CI: 0.764–1.060), bladder exstrophy (PRR = 1.039, 95% CI: 0.634–1.704), and conjoined twins (PRR = 0.623, 95% CI: 0.293–1.326) remained stable.

### Correlation between important characteristics in early pregnancy and BDs

Table 6 shows the correlations between important demographic characteristics in early pregnancy and BDs. Notably, BDs were positively correlated with pregnant women's age (*R* = 0.732, *P* < 0.01) and education level (*R* = 0.586, *P* < 0.05) and having PGDM/GDM (*R* = 0.711, *P* < 0.01), while when the pregnant women had a family history of a dead fetus (*R* = −0.536, *P* < 0.05) and a birth of a fetus with BDs (*R* = −0.528, *P* < 0.05) were negatively correlated with BDs.

**Table 6:** Correlation between important characteristics in early pregnancy and birth defects.

Factors	*R*	*P*-value
Maternal age	0.732	<0.01
Gravidity	−0.014	>0.05
Parity	−0.496	>0.05
Education level	0.586	<0.05
Family monthly income per capita	0.300	>0.05
Fever	−0.290	>0.05
Viral infection	0.079	>0.05
PGDM/GDM	0.711	<0.01
Antibiotic use	0.064	>0.05
Contraceptive drug use	−0.062	>0.05
Sedative use	−0.216	>0.05
Alcohol use	−0.273	>0.05
Pesticide exposure	0.150	>0.05
Radiation exposure	−0.350	>0.05
Chemical exposure	0.025	>0.05
History of dead fetus	−0.536	<0.05
History of spontaneous abortions	0.214	>0.05
History of BDs	−0.528	<0.05
History of consanguineous marriage	−0.102	>0.05

BDs, birth defects; PGDM, pre-gestational diabetes mellitus; GDM, gestational diabetes mellitus.

## Discussion

The data in this study described the cases of BDs enrolled in GXBDMN between 2016 and 2020, and 25 types of BDs were identified by the criteria of ICD-10. This is the first study to perform univariate Poisson regression to calculate the PRR to explore the changing trends of BDs prevalence in Guangxi of China. Furthermore, the causes of BDs in Guangxi were also reported, so a comprehensive analysis of the potential associated risk factors of BDs in this study was conducted. The study would help the public health policy-makers as well as individuals take preventive measures beforehand to reduce BDs and infant deaths as well as contribute to public health interventions by setting strategies, organizing resource planning by legislators, and informing the parents.

In this study, the overall prevalence of BDs in Guangxi between 2016 and 2020 (121.71 per 10,000 PIs) was only slightly higher than 121.46 per 10,000 PIs, which was reported in a study of comprehensive prevention and control effect on BDs in Guangxi between 2000 and 2015 (27). Between 2000 and 2015, the prevalence of BDs in Guangxi has a decreasing trend: the BDs prevalence during 2000–2003 and 2004–2009 increased from 201.80 to 207.13 per 10,000 PIs, and then during 2010–2015, it decreased to 112.60 per 10,000 PIs by a large margin (27). However, in this study, between 2016 and 2020, the prevalence of BDs in Guangxi increased year by year from 103.06 (95% CI: 100.92–105.20) to 155.36 (95% CI: 152.17–158.55) per 10,000 PIs, with a PRR of 1.116; the prevalence of live birth BDs in Guangxi also increased year by year from 88.12 (95% CI: 86.13–90.10) to 145.58 (95% CI: 142.48–148.68) per 10,000 live birth PIs, with a PRR of 1.143 (95% CI: 1.135–1.151). Therefore, in the past two decades, the prevalence of BDs in Guangxi first decreased from 2000 to 2015 and then increased from 2016 to 2020. It was reported that the prevalence of BDs in China between 2017 and 2021 was 2.50% (15) and much higher than the BDs prevalence in Guangxi in this study. This changing trend of BDs prevalence in Guangxi in the past two decades was similar to Henan, which is a province in China. The prevalence of BDs in Henan between 1997 and 2019 was 122.50 per 10,000 PIs, the BDs prevalence decreased from 109.80 per 10,000 PIs in 1997 to 80.19 per 10,000 PIs in 2011 (*P*-trend < 0.05) and then increased to 235.70 per 10,000 PIs in 2019 by a large margin (*P*-trend < 0.05) (28). Therefore, this changing trend of BDs prevalence in Guangxi is very important, and the government needs to pay more attention to it in the prevention and control policy-making process.

CHDs have become a big and serious public health problem in the world and in China in recent years. Several studies have reported that CHDs are the most common type of BDs seen so far in China, and the prevalence of CHDs increases significantly by year (15, 16, 29). In some other provinces of China, such as the prevalence of CHDs in Henan province even up to 136.46 per 10,000 PIs in 2019 (28), which was nearly four times the CHDs prevalence of Guangxi (35.82 per 10,000 PIs) in the same year. In this study, the prevalence of CHDs also had a significant increase throughout the study period. This might be explained by the continuous improvement of the BDs surveillance system and the development of diagnostic technology for CHDs. Especially the gradual improvement of integrated prenatal and postpartum screening and diagnostic networks for BDs, as well as the continuous improvement of screening and diagnostic capabilities, which leads to the increased possibility of diagnosing asymptomatic and mild CHDs patients (30). Therefore, the health management department needs to highly focus on the changing trends of CHDs to make a good response strategy.

One review report has indicated that the differences in the dosage of X-linked genes lead to a sex bias in gene expression, which has a higher susceptibility to specific diseases of the Y chromosome than the X chromosome (31). Then, a national population-based study in the United Kingdom confirms that male PIs were more likely to be born with major BDs (32). The univariate Poisson regression model in this study showed that the prevalence of BDs in male PIs was significantly higher than that in female PIs (PRR = 1.289, *P* < 0.05). This result is consistent with other scholars’ study results (13, 33–36). Moreover, the study by Zhou et al. (13) showed that PIs of women who conceived in summer (June, July, August) had the lowest risk of having BDs, while PIs of women who conceived in spring (March, April, May) or winter (December, January, February) had a higher risk of having with BDs. This might be explained by the abundance of rich and varied fresh vegetables and fruits in summer that are beneficial to the health of pregnant women and their fetuses (13). Therefore, it is important for women to conceive at the most appropriate time (13). However, because the BDs prevalence was calculated by the time of giving birth (quarters), the results in this study might be different from the study by Zhou et al. (13). In this study, the univariate Poisson regression model showed a significant difference in the prevalence of BDs in different quarters (seasons). PIs who were born in the fourth quarter (October, November, December) had the lowest risk to have BDs, while PIs who were born in the second quarter (April, May, June) had the highest risk to have BDs. The above research results suggest that the burden of BDs on male infants is higher than that on female infants, and an appropriate conception time can relatively reduce the chance of infants to be born with BDs. Therefore, health management department should focus on the different distribution of BDs prevalence between males and females and provide maternal healthcare services to pregnant women for the prevention of BDs and more prenatal BDs counseling for women who prepare for pregnancy, to effectively reduce the chance of BDs in infants.

It is well known that BDs can be influenced by multiple factors (33, 37). Although it is not exactly known what causes most BDs, some associated risk factors might make pregnant women more likely to have a fetus with BDs, such as changes in genes or chromosomes, environmental factors, certain health conditions, taking certain medicines before or during pregnancy, smoking and drinking alcohol during pregnancy, getting certain infections during pregnancy, and age (38). These factors might play a key role in the increased risk of having a fetus with BDs (38). It has been evident that BDs might be influenced by maternal age (34) and education level (39), illness during early pregnancy (40), and family history of the disorder (12). Maternal age should be taken into account when analyzing the potential risk factors of BDs. In many studies, it has been confirmed to be a risk factor for BDs (34, 41). Maternal age of ≥35 years can increase the risk of fetuses born with BDs compared with those aged <35 years [odds ratio (OR) = 2.09, 95% CI: 1.13–3.85] (41). In this study, it has also been confirmed that BDs were positively correlated with pregnant women's age. Older pregnant women were more likely to have a fetus with BDs, especially those aged ≥35 years. This might be explained by the maternal age-related anomalies following China's new two-child policy, especially that the proportion of advanced maternal age (35 years and older) significantly increases after China implemented a universal two-child policy (since 2016) (42). However, a meta-analysis showed that there was a low-quality evidence suggested that pregnant women in older age have an increased risk of having fetuses with BDs (43). The finding indicates that high-quality and targeted counseling is needed before perinatal management, especially for advanced maternal age.

Moreover, the maternal educational level should be considered when exploring the risk factors of BDs. However, it is well known that there is no consensus on whether the high maternal education level is a risk factor or a protective factor for BDs. Most scholars indicate that low maternal educational level is a risk factor for BDs (39, 44), because it can significantly increase the risk of having a fetus with BDs (OR = 8.40, 95% CI: 2.17–32.52) (45), but other researchers find that high maternal educational level is also a risk factor for BDs, such as DS (11). In this study, it is observed that pregnant women with high education levels were more likely to give birth to a fetus with BDs. Therefore, although this study showed that pregnant women with high education levels were also a risk factor for BDs, there is still a need to increase awareness about BDs among rural women before pregnancy, those with unplanned pregnancies, and those who have lower educational levels.

In addition, pregnant women with PGDM before pregnancy or GDM in early pregnancy have been confirmed to have a risk of giving birth to an offspring with BDs (40, 44, 46). Regardless of being obese, pregnant women with PGDM are strongly associated with most types of BDs (OR ranging from 2.0 to 75.9) (46). Approximately 12.7% to 14.8% of pregnant women with PGDM will bear a fetus with BDs (47, 48). It is reported that pregnant women with GDM is an increased risk of overall BDs prevalence in offspring born [relative risk (RR) = 1.18, 95% CI: 1.13–1.23] (40). By comparing various potential risk factors of BDs, this study also found that pregnant women with PGDM before pregnancy or with GDM in early pregnancy were positively correlated with BDs. Hence, screening diabetes for pregnant women is a better identification of offspring at risk for BDs, especially that better glycemic control requires pregnant women to pay more attention to their dietary habits and nutritional supplementation during pregnancy.

Finally, some interesting results were also found in this study. Pregnant women who had a family history of giving birth to a dead fetus or giving birth to a fetus with BDs was a risk factor for BDs. Therefore, pregnant women need to pay more attention to prenatal screening and diagnosis after knowing that they have a family history of BDs. It is widely known that fetal death is one of the adverse consequences of BDs (16). A family history of fetal birth in pregnant women is generally identified as a risk factor for BDs, which will greatly increase the probability of PIs with BDs (OR = 3.84, 95% CI: 1.64–8.96) (12, 49). Especially, having three or more PIs with BDs is a stronger risk factor of a family history of BDs (49). Although pregnant women with adverse pregnancy outcome history were found to be an independent protective factor of BDs in this study, such as a history of previous fetal abnormalities and fetal death. With this, it is still necessary to proceed with prenatal diagnosis and develop scientific pregnancy preparation plans for high-risk pregnant women, especially those with a history of adverse pregnancy outcomes.

## Strengths and improvements

There are two major strengths of this study. First, the BDs surveillance data of this study were based on BDs monitoring hospitals throughout Guangxi, the epidemiological characteristics of BDs, and potential associated risk factors of BDs over the study period that can be accurately presented. Second, this paper was the first study based on the Poisson regression model to explore the changing trends of BDs prevalence in Guangxi, especially that the comprehensive analysis of the associated risk factors of BDs in this study is very helpful in the future study. Therefore, this study will help the government further understand the changing trends and causes of BDs in the future and can help provide strong evidence for the government during the formulation of public health policies.

However, the BDs data in this study were obtained from GXBDMN, which was a hospital-based and passive BDs surveillance system. There might be a slight deviation in the risk factor analysis results based on GXBDMN. Therefore, it is crucial to further improve the surveillance methods, detection strategies, and screening methods for the population with BDs in the future. For example, in addition to recording the various indicators required by the BDs monitoring scheme, it can also record various physical changes, medication taken, dietary habits, environmental exposure (such as radiation exposure and pesticide exposure), and psychological changes during the entire pregnancy cycle of pregnant women. Establishing a population-based cohort to study BDs might be a good solution in the future.

## Conclusions

A significant increase in the prevalence of BDs was detected between 2016 and 2020 in Guangxi of China. CHDs were the most common BDs in Guangxi, accounting for about 35% of all BDs types by 2020, followed by polydactyly, especially that the prevalence of CHDs most significantly increased through the study period. Different risk factors can inﬂuence BDs development. In particular older maternal age, higher maternal education level, and having PGDM before pregnancy or GDM in early pregnancy were the risk factors that can influence BDs. It is more likely to bear a fetus with BDs in those pregnant women. These findings can be used to support future research and the planning of BDs prevention and control strategies, to better provide more resources and prenatal screening services to pregnant women and to those who are planning to conceive to prevent BDs. Moreover, although the results of this study elucidated some related risk factors for BDs from a methodological perspective, there is still room for improvement. For example, the inclusion of risk factors related to BDs in association analysis is relatively rare due to the monitoring methods of the monitoring system, which may result in a slight bias in this study. Therefore, developing a prospective BD cohort of all gestational weeks and multiple factors based on pregnant women will be the key direction of research in future studies. This cohort will be able to further validate the results of this study, providing strong scientific and technological support for the prevention and control of BDs.

## Data Availability

The original contributions presented in the study are included in the article, further inquiries can be directed to the corresponding authors.
